# Novel Evidence for the Increasing Prevalence of Unique Names in China: A Reply to Ogihara

**DOI:** 10.3389/fpsyg.2021.731244

**Published:** 2021-12-06

**Authors:** Han-Wu-Shuang Bao, Huajian Cai, Yiming Jing, Jianxiong Wang

**Affiliations:** ^1^Institute of Psychology, Chinese Academy of Sciences, Beijing, China; ^2^Department of Psychology, University of Chinese Academy of Sciences, Beijing, China; ^3^School of Economics, Beijing International Studies University, Beijing, China

**Keywords:** name, uniqueness, individualism, cultural change, China

## Abstract

In this study, we aimed to address three comments proposed by Ogihara on a recent study where we found that unique names in China have become increasingly popular from 1950 to 2009. Using a large representative sample of Chinese names (*N* = 2.1 million), we replicated the increase in uniqueness of Chinese names from 1920 to 2005, especially since the 1970s, with multiple uniqueness indices based on name-character frequency and name-length deviation. Over the years, Chinese characters that are rare in daily life or naming practice were more often used in given names, and the length of given names became more deviant from typical practice (i.e., more one-character and three-character given names and higher standard deviation of name length). Taken together, these findings not only reconfirmed the increasing prevalence of unique names but also demonstrated the validity of various indices in assessing name uniqueness in China.

## Introduction

Over the past decades, massive sociocultural changes have occurred around the world. As a special case, shifts in naming practices have received much attention. The selection of unique names for children has become increasingly popular and represents a widely observed phenomenon. For instance, research has revealed that the tendency of giving children a unique name has increased in the United States from 1880 to 2015 (Twenge et al., 2010, 2016), in the United Kingdom from 1838 to 2016 (Bush et al., 2018), in Germany from 1894 to 1994 (Gerhards and Hackenbroch, 2000), and in Japan from 2004 to 2018 (Ogihara et al., 2015; Ogihara, 2021a).

Relevant to our current paper, Cai et al. (2018) examined the tendency toward unique name selection in China. In that study, they sampled 600 Chinese names, with 10 names for each year between 1950 and 2009. By referring to the character frequency in daily use (the “Modern Chinese Character Frequency of Use Dictionary”; i.e., a corpus of contemporary Chinese characters used in daily life), they obtained the average frequency of characters as an index of name uniqueness, with lower frequency indicating higher uniqueness. The results showed that Chinese have been more likely to use unusual Chinese characters to name their children from 1950 to 2009 (Cai et al., 2018). Thereby, they concluded that unique names have become more popular in China over the past decades. Since the publication of this study in 2018, it has been widely cited (more than 45 citations).

Recently, however, this study was challenged (Ogihara, 2020). In a comment paper, Ogihara (2020) cast doubt on the validity of the name uniqueness index, and furthermore, the persuasiveness of the findings. In particular, he proposed three questions.

First, he questioned whether using the frequency of Chinese characters in *daily life* to indicate the uniqueness of Chinese name characters was appropriate. A character that is rare in daily use may not be equally rare in naming practices (e.g., *Hao*, in Chinese, “昊”). Moreover, the popularity of name characters may vary with the cohort (Ogihara, 2020). Therefore, he thought that name-character frequency based on a database of characters used in *baby names* for each cohort would be more appropriate.

Second, he wondered how the length of Chinese names (i.e., the number of Chinese characters in a name) has changed over time. A Chinese given name may consist of one, two, or even three characters. Compared with a single-character given name, a given name with multiple characters is more likely to be distinct from other names (i.e., to be unique). That is, name length is also a potential index of name uniqueness in China, with a longer name indicating higher name uniqueness (He et al., 2021). Hence, examining the change in name length deserves consideration.

Third, he wondered how the pronunciation of Chinese names has changed. In examining the change of name uniqueness in Japanese names, Ogihara found that it was the pronunciations, rather than characters or character combinations, of Japanese names that have become increasingly unique (Ogihara et al., 2015; see also Ogihara, 2021b). Accordingly, he wondered whether this is also the case in China.

In this study, we addressed these questions except for the one related to pronunciation of name characters. We did not think pronunciation of name characters is as meaningful in China as in Japan, because Chinese characters used in Chinese language differ substantially from those used in Japanese, especially in pronunciation. A Chinese character in Chinese usually has a single fixed pronunciation, whereas most Chinese characters in Japanese carry multiple pronunciations based on context or usage (Ogihara, 2020, 2021b). Moreover, in contrast to the situation in Japan, one pronunciation in China may refer to multiple Chinese characters that differ in many aspects, including meaning and uniqueness. Therefore, name pronunciation should not be a valid index of name uniqueness in China.

We utilized a large representative sample of more than 2 million Chinese names over a broader time span (1920∼2005). First, we tested two indices of name uniqueness based on *name-character frequency*: one was identical as we had used in our previous study (i.e., character uniqueness in daily use; Cai et al., 2018) and the other was the alternative one suggested by Ogihara (i.e., character uniqueness in naming practice; Ogihara, 2020). We examined whether we could replicate the rising trend of unique names with these two indices.

**FIGURE 1:**
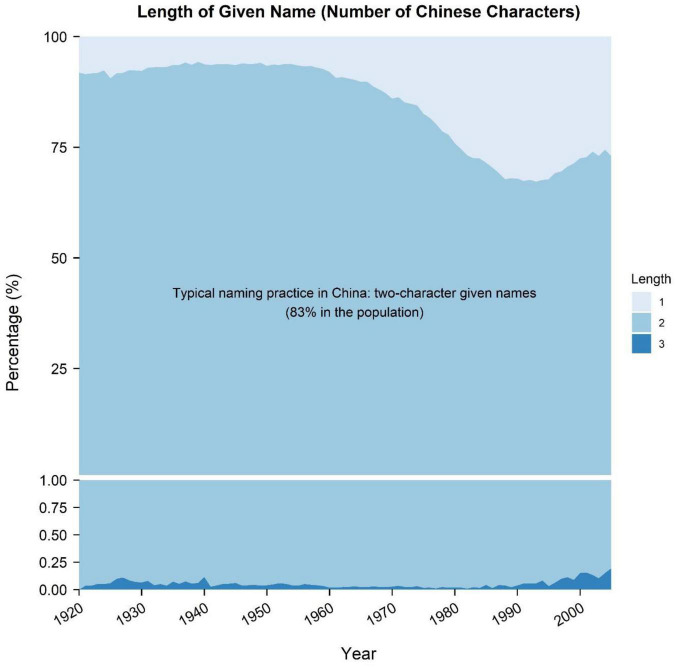
Trends of the percentages of one-, two-, and three-character given names.

Then, we tested four indices based on *name-length deviation*. Besides the absolute (average) length of given name as suggested by Ogihara (2020), we also tested three other potential indices of given name for a certain year: proportion of one-character given name, proportion of three-character given name, and standard deviation of the length of given name. Traditionally, the majority of Chinese given names consist of two characters: one represents the generation in a family and the other denotes the unique identity of a person (Zhu and Millward, 1987; see also Figure 1). Since “[d]eviating from typical practice is one way to express uniqueness” (Ogihara, 2020, p. 2), a given name deviating from typical two-character given names (i.e., one-character or three-character names; Han Chinese given names at most contain three characters) may suggest atypical or unique. Therefore, the proportion of one-character and three-character given names may serve as indices of name uniqueness. Moreover, the diversity of name length as indicated by the standard deviation of name length may also serve as an index of name uniqueness.

In summary, we would test whether unique names in China have been on the rise with a large representative sample of Chinese names and six potential indices of name uniqueness. In doing this, we hope to clarify the concerns proposed by Ogihara (2020).

## Methods

### Sample

To obtain a nationally representative sample of Chinese names covering a long period, we accessed data from the 2005 China’s 1% Population Census (National Bureau of Statistics [NBS] of China, 2005). The 2005 China Census was conducted using a three-stage stratified cluster sampling method, with respondents randomly selected from each of 340 prefectural-level cities or regions in China. Our sample was a random subset (*N* = 2,585,481) drawn by the NBS, which had been widely used in previous economic and population research.

The 2005 China Census collected respondents’ surname, given name, gender, birth year, ethnicity, and several other demographic and household variables. Because 89.1% of Chinese are Han Chinese and the naming norms are quite different between Han Chinese and ethnic minorities, we restricted our sample to Han Chinese only. Moreover, because the sample sizes for birth years < 1920 were not sufficient, we limited the range of birth years to 1920∼2005. Finally, we scrutinized the dataset and excluded those who did not have formal names (e.g., recorded as “unnamed”). The final sample consisted of 2,144,025 individuals (50.3% male). The sample size of each year ranged from 2,178 to 48,152 (*Median* = 26,514).

### Indices of Name Uniqueness

We used six indices to measure name uniqueness. Two were based on the frequency of Chinese characters in a given name, whereas the other four were derived from the length of a given name (i.e., the number of Chinese characters in a given name).

#### Indices Based on Name-Character Frequency

##### Character-Corpus Uniqueness (in Daily Use)

Since most characters used in Chinese given names can be used in daily contexts, the uniqueness of a character can be estimated according to a contemporary Chinese corpus^1^ (e.g., the corpus we used here). The previous study used such “character-corpus uniqueness” (CCU) to indicate name uniqueness (Cai et al., 2018, Study 2). We tested this index to check the robustness of the previous findings (Cai et al., 2018). The percentage of each character in the Chinese corpus was log-transformed (a small constant 10^–6^ was added to adjust for its positive skewness) and reversed:


CCU=-log(P+character[incontemporaryChinesecorpus]10)-610.

CCU ranges from 1.3 to 6. For example, CCU = 2 and 3 mean that the frequency of a character in the Chinese corpus equals to 1/100 and 1/1,000, respectively.

##### Name-Character Uniqueness (in Naming Practice)

To compute name-character uniqueness in naming practice for each individual in the 2005 China Census, we accessed a Chinese name database from the National Citizen Identity Information Center (NCIIC) of China. This database is publicly available in the R package “ChineseNames” (Bao, 2021), which consists of nationwide frequencies of 1,806 Chinese surnames and 2,614 Chinese characters used in given names, covering about 1.2 billion Han Chinese (96.8% of the Han Chinese household-registered population born between 1930 and 2008 and still living in 2008). Percentages of people whose given names included each of the 2,614 name characters were documented separately for six birth cohorts (pre-1960s, 1960∼1969, 1970∼1979, 1980∼1989, 1990∼1999, and 2000∼2008).

To account for changes in the popularity of specific given names over time (Grossmann and Varnum, 2015; Ogihara, 2020), we estimated name-character uniqueness (NU) based on the percentage of a name character used in the Han Chinese population within a specific birth year (*P*_character_)—an approximate estimate for a birth year using the weighted character frequencies of the nearest two birth cohorts (for computational details, see the “compute_name_index” function in the R package “ChineseNames”; Bao, 2021). Then, we log-transformed *P*_character_ and reversed it:


NU=-log(P+character[innamingforabirthyear]10)-610.

NU ranges from 1.2 to 6. For instance, NU = 2 and 3 mean that 1/100 and 1/1,000 of people in their birth year used this character in given name, respectively.

#### Indices Based on Name-Length Deviation

As aforementioned, we derived four indices for each year from the length of given name: (1) proportion of one-character given name, (2) proportion of three-character given name, (3) absolute (average) length of given name, and (4) standard deviation of name length. To note, in the 2005 China Census sample we used here, 82.82% of Han Chinese possessed two-character given names, whereas only 17.14 and 0.04% held one-character and three-character given names, respectively.

## Results

**FIGURE 2:**
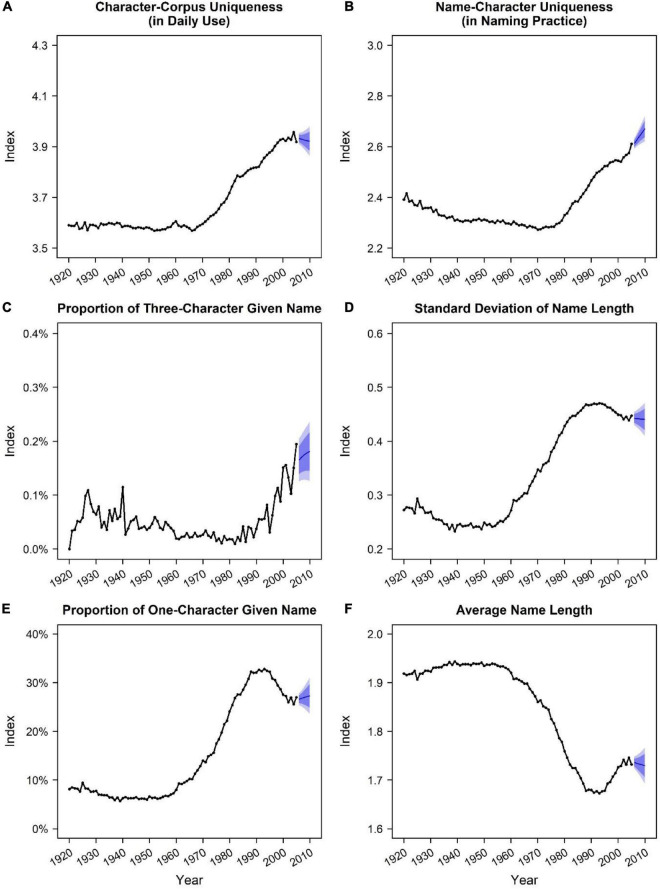
Trends of six name indices in China (1920∼2005). We used the R package “forecast” to add potential changes from 2006 to 2010, as shown in blue lines with prediction intervals at 80 and 95% confidence levels. **(A)** Character-corpus uniqueness (in daily use) by year. **(B)** Name-character uniqueness (in naming practice) by year. **(C)** Proportion of three-character given name by year. **(D)** Standard deviation of name length by year. **(E)** Proportion of one-character given name by year. **(F)** Absolute (average) name length by year.

Figure 2 presents how the six name indices have changed in China. We regressed each index on birth year to estimate their annual changes (*b_year_*) in different periods. The two indices based on name-character frequency manifested similar changing patterns. Before 1970, the annual changes of character-corpus uniqueness (*b_year_* = –0.00020, *SE* = 0.00009, *t* = –2.30, *p* = 0.026) and name-character uniqueness (*b_year_* = –0.00204, *SE* = 0.00014, *t* = –14.80, *p* < 0.001) were trivial. However, since 1970, both character-corpus uniqueness (*b_year_* = 0.0105, *SE* = 0.0003, *t* = 34.41, *p* < 0.001) and name-character uniqueness (*b_year_* = 0.0104, *SE* = 0.0003, *t* = 37.51, *p* < 0.001) have linearly and continuously increased at a steady speed (Figures 2A,B). Together, both frequency-based indices indicated an overall rising trend of unique names in China from 1920 to 2005, particularly after 1970.

Similarly, two of four indices based on name-length deviation indicated that Chinese naming practices have increasingly deviated from the typical naming practice of choosing two-character names for children. Specifically, the percentage of three-character given name did not significantly change from 1920 (0.00%) to 1959 (0.03%) (*b_year_* = –0.0004, *SE* = 0.0003, *t* = –1.47, *p* = 0.15) and from 1960 (0.02%) to 1989 (0.02%) (*b_year_* = 0.0001, *SE* = 0.0002, *t* = 0.38, *p* = 0.71) but increased sharply from 1990 (0.04%) to 2005 (0.19%) (*b_year_* = 0.0089, *SE* = 0.0013, *t* = 6.70, *p* < 0.001) (Figure 2C); the standard deviation of name length decreased trivially from 1920 (*SD* = 0.27) to 1959 (*SD* = 0.26) (*b_year_* = –0.00084, *SE* = 0.00015, *t* = –5.68, *p* < 0.001), increased largely from 1960 (*SD* = 0.27) to 1989 (*SD* = 0.47) (*b_year_* = 0.0073, *SE* = 0.0002, *t* = 47.11, *p* < 0.001), and decreased negligibly to a moderately high level from 1990 (*SD* = 0.47) to 2005 (*SD* = 0.45) (*b_year_* = –0.0022, *SE* = 0.0002, *t* = –10.05, *p* < 0.001) (Figure 2D).

Notably, the other two of four indices based on name length—the percentage of one-character given name (Figure 2E) and the absolute (average) name length (Figure 2F)—manifested almost reverse patterns with each other. The percentage of one-character given name showed a similar changing pattern with the standard deviation of name length (i.e., first decreasing trivially, then increasing largely, and finally shifting to a moderately high level). In contrast, shifts in average name length were almost opposite to that pattern. This finding was not a surprise. As could be seen in Figure 1, compared with the increase in one-character names (from about 10 to 30%), the increase in three-character names (from about 0.05 to 0.20%) was neglectable. Thus, the overall declining trend of average name length was indeed primarily driven by the overall rising trend of one-character given names. Given that the vast majority of Han Chinese possessed two-character given names, these results may also denote that atypical naming practices became more prevalent in China, though the interpretations and implications of these two indices were more complex than the other indices.

In summary, the name-uniqueness indices based on name-character frequency and name-length deviation produced similar findings: an increasing trend of name uniqueness in China over the past decades, especially since the 1970s.

## Discussion

Research has demonstrated increasing prevalence of unique names in many countries. In a widely cited study, Cai et al. (2018) found this is also the case in China. Ogihara (2020), however, doubted the validity of both the index used and the findings in that study. In the present study, we clarified his concerns by empirically replicating our previous findings and testing his suggestions (Ogihara, 2020). We used an unprecedentedly large representative sample of Chinese names, covering a longer period of time from 1920 to 2005. We believe that we have achieved our goal.

Ogihara (2020) questioned both the appropriateness of using a character corpus in daily life to compute the frequency of name characters in a name as well as the findings based on the corpus. We believe that this methodology, albeit not perfect, is valid: people may consider a name to be unique if the name characters are rare in daily use. Indeed, we found a rising prevalence of unique names since 1950 as similarly shown in our previous study (Cai et al., 2018). Ogihara (2020) also suggested a new way to estimate name uniqueness by referring to the frequency of characters used in naming practices in a certain birth cohort. We also believe that this methodology is valid: people may consider a name to be unique if the name characters are unusual in people’s names. We tested this index and obtained a similar finding. Overall, the two uniqueness indices based on character frequency yielded similar results, suggesting that both are valid because both tap name uniqueness in specific ways.

Ogihara (2020) further suggested name length may be relevant to name uniqueness in China. We tested absolute name length as he suggested and three other length-based indices: the proportion of one-character given name, the proportion of three-character given name, and the standard deviation of name length. Although the specific trends of these indices were somewhat distinct from each other, all indices yielded findings that indicate an overall rising trend of deviating from the most typical naming practice in China (i.e., giving two-character names to children). Notably, it is not the low probability to be duplicated that serves the underlying logic for absolute name length to be indicative of name uniqueness; instead, it is the high probability of deviation from typical naming practice that makes a difference, in our case, the increase in one-character given names (rather than three-character given names) as we explained in the results.

Taken together, we have replicated our previous finding, the rising trend of unique names in China, with two indices based on name-character frequency and four indices based on name-length deviation. Thus, we have reconfirmed the rising trend of name uniqueness in China and clarified the concerns proposed by Ogihara (2020).

Our research also contributes to the existing literature in several other ways. To our knowledge, this is the first study that uses multiple indices of name uniqueness in studying the temporal change of Chinese names. The convergent findings suggest that all six indices are valid to some extent and therefore applicable in future relevant studies. What we need to be aware is that each of these indices approaches the uniqueness of name in different ways, capturing either a specific kind of infrequency of name character or a specific kind of deviation from typical naming practice. To some extent, they are complementary to each other because all of them have captured some unique aspects of name uniqueness. If only one index is needed in a study, however, we would not recommend the absolute name length because its implication is unclear, depending on the relative importance of one-character names over three-character names or vice versa. Instead, *name-character uniqueness in naming practice* is preferred. This index is easy to understand and to be computed using the R package “ChineseNames” (Bao, 2021). More importantly, unlike character uniqueness in daily use, it takes birth cohort information into account so that the estimation would be more accurate; and unlike other length-based indices that only apply to group-level analyses, it also allows individual-level analyses because the uniqueness of each individual’s name can be calculated.

Moreover, our research advances a better understanding of cultural changes in China. A vast literature has used cultural products such as word usage in Google Books to reflect individualist values, revealing an *increasing* trend of individualism in China over the past decades (for reviews, see Cai et al., 2019; Kashima et al., 2019). Somewhat unexpectedly, there is also research showing a *decreasing* trend of individualist values in China from 1990 to 2007 (Santos et al., 2017). Since unique naming is a cultural practice that is theoretically associated with individualism (Twenge et al., 2010; Grossmann and Varnum, 2015; Ogihara et al., 2015; Ogihara, 2021a), the findings from our previous research (Cai et al., 2018) and our current replication study together suggest that the cultural emphasis on uniqueness is on the rise—a manifestation of increasing individualism in China.

## Data Availability Statement

The raw data supporting the conclusions of this article will be made available by the authors, without undue reservation.

## Ethics Statement

The studies involving human participants were reviewed and approved by Institute of Psychology, Chinese Academy of Sciences. Written informed consent for participation was not required for this study in accordance with the national legislation and the institutional requirements.

## Author Contributions

HC proposed, designed the research, and revised the manuscript. H-W-SB and JW collected the data. H-W-SB analyzed the data and drafted the manuscript. YJ provided critical feedback. All authors approved the submitted version of the manuscript.

## Conflict of Interest

The authors declare that the research was conducted in the absence of any commercial or financial relationships that could be construed as a potential conflict of interest.

## Publisher’s Note

All claims expressed in this article are solely those of the authors and do not necessarily represent those of their affiliated organizations, or those of the publisher, the editors and the reviewers. Any product that may be evaluated in this article, or claim that may be made by its manufacturer, is not guaranteed or endorsed by the publisher.

## Footnotes

1 The corpus we used in this study was retrieved 16 November 2016 from http://www.cncorpus.org (a modern Chinese corpus by the National Language Committee of China). However, this link is now invalid, so we made the character frequencies available in the R package “ChineseNames” (Bao, 2021).
